# Engineering Antibody Evolution: Germline‐Targeting HIV Vaccines Reach a Turning Point

**DOI:** 10.1111/imcb.70169

**Published:** 2026-10-06

**Authors:** Rowena A. Bull, Deborah L. Burnett

**Affiliations:** ^1^ School of Biomedical Sciences UNSW Sydney Kensington New South Wales Australia; ^2^ The Kirby Institute UNSW Sydney Kensington New South Wales Australia; ^3^ Garvan Institute of Medical Research Darlinghurst New South Wales Australia

## Abstract

This commentary examines three recent *Nature* studies demonstrating a conceptual shift in HIV vaccine design, demonstrating how germline‐targeting HIV vaccines can progress beyond expanding rare broad neutralizing antibody (bnAb) precursors to deliberately guiding their affinity maturation into bnAbs in non‐human primates.

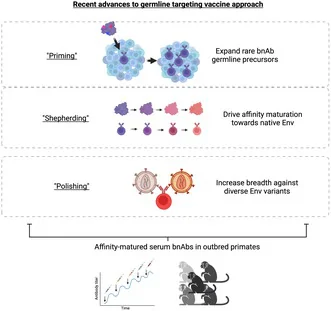

Despite more than four decades of intensive research on human immunodeficiency virus‐1 (HIV‐1), no protective vaccine has been licensed. The extraordinary genetic diversity of HIV‐1 is a central barrier to the development of a broadly protective vaccine. Broadly neutralizing antibodies (bnAbs) against conserved envelope (Env) epitopes can arise naturally but only in a small subset of infected individuals and typically take years to develop [1]. These bnAb precursors exhibit low initial affinity for native HIV Env, requiring extensive somatic hypermutation (SHM) to develop neutralizing potency. In addition, many classes of bnAb precursors are present at low frequencies and contain unusual features, such as long heavy‐chain complementarity‐determining region 3 (HCDR3) loops that are associated with autoreactivity [2] (Figure 1A). Conventional Env immunogens have largely failed to reliably elicit bnAbs, instead preferentially eliciting strain‐specific or non‐neutralizing antibodies. Germline‐targeting vaccine strategies have emerged as a solution to this problem. Rather than directly eliciting mature bnAbs, germline‐targeting vaccines address this problem in stages: priming immunogens engage and expand rare naïve precursor B cells while sequential heterologous boosters guide their affinity maturation towards broad neutralization [2]. Phase I clinical trials have demonstrated reproducible priming of some bnAb precursors, including the VRC01‐class [3], while preclinical studies have demonstrated that other bnAb precursor classes, including those targeting the V2‐apex and V3‐glycan, can also be recruited in non‐human primates [4, 5]. However, whether vaccination can reproducibly shepherd these precursors through the prolonged affinity maturation required to generate serum nAbs at sufficient breadth and protective titres remained unresolved.

**FIGURE 1:**
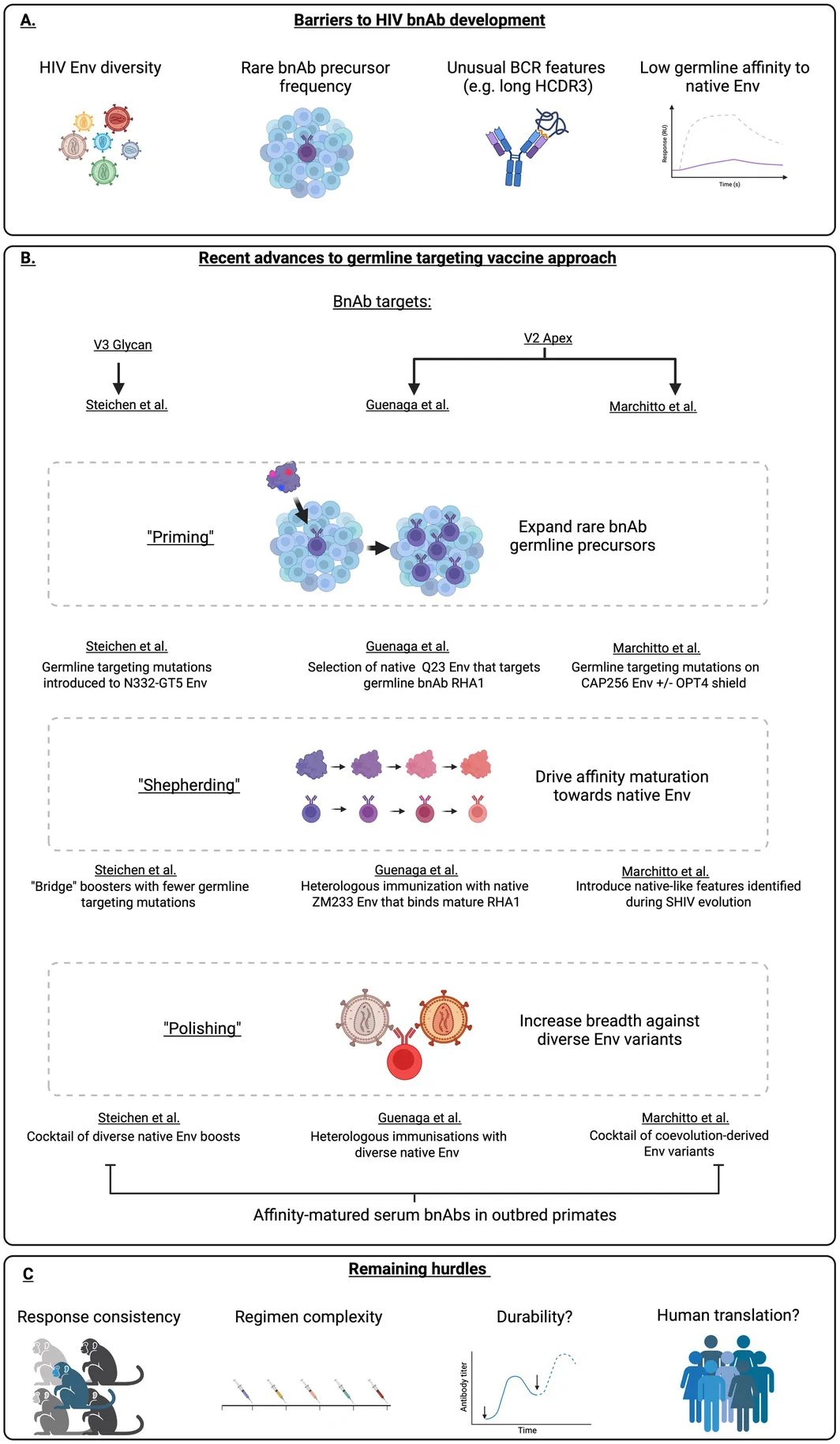
Design principles, comparative approaches and remaining challenges for germline‐targeting HIV vaccines. (A) Core barriers to HIV bnAb development that germline‐targeting vaccine approaches aim to overcome, including the breadth required to span across HIV virion diversity and the challenges of inducing bnAb precursors due to their rarity, low affinity, and unusual antibody features. (B) Comparison of recent publications presenting sequential immunization strategies [6, 7, 8] to guide V3‐glycan and V2‐apex bnAb lineages through priming, shepherding, and polishing. (C) Key remaining translational hurdles, including prolonged immunization schedules, species‐specific precursor repertoires, variable response magnitude, and uncertain durability.

Three recent *Nature* studies now provide compelling evidence from non‐human primates that sequential heterologous vaccinations can guide rare precursors to become bnAbs [6, 7, 8]. Although the studies targeted two different conserved epitopes and employed distinct immunogen‐design strategies, they collectively demonstrated a fundamental shift in HIV vaccine design, from engineering antigen recognition to engineering antibody evolution.

A key aspect of germline‐targeting approaches is the initial engagement of rare bnAb precursors with low‐affinity to native Env (Figure 1B). As such, the “priming” antigens are either designed or selected to engage and expand these rare bnAb precursors. Both Steichen et al. and Marchitto et al. rationally designed antigens for optimal precursor engagement. Steichen's V3‐glycan immunogen, N332‐GT5, incorporates twenty engineered mutations that specifically enhance binding of V3‐glycan targeting, rare BG18‐class precursors. Marchitto introduced germline‐targeting mutations into the CAP256 superinfecting founder to generate CAP256.OPT4, further introducing variants with engineered mutations to shield immunodominant off‐target sites to maximize precursor engagement of V2‐apex targeting bnAbs. By contrast, Guenaga et al. took a non‐standard approach compared to traditional germline‐priming antigens; instead of engineering the priming antigen, they screened native Env trimers for their ability to bind the inferred precursors of the V2‐apex bnAb RHA1, identifying Q23 as a naturally compatible immunogen. All three priming strategies successfully engaged and expanded multiple different bnAb precursors, both within individual macaques and across the cohorts.

After successfully priming germline bnAb precursors, all three studies used heterologous immunisations not only to maintain the recruited bnAb lineages but also to “shepherd” them through repeated rounds of selection and affinity maturation towards native Env. Steichen immunized with a series of subsequent immunogens with progressively fewer engineered mutations before ultimately presenting diverse native Env trimers. Marchitto used viral escape pathways observed during chronic SHIV infection to design the subsequent immunogens. Marchitto further showed that the antigenic features favoring precursor recruitment differed from those promoting breadth: positively charged V2‐loops enhanced precursor engagement, whereas evolution towards negatively charged V2‐loops, together with changes in V1 architecture and glycosylation, were associated with neutralization breadth. For shepherding, Guenaga again differed in that heterologous immunisations used a single native Env trimer rather than sequentially evolving antigens. Here, they used native ZM233 Env, selected for its ability to bind affinity‐matured RHA1 rather than the germline precursor.

Finally, all three studies used heterologous cocktails of diverse native Env to “polish” and increase the breadth of their affinity‐matured bnAbs. These Env trimers were designed to target and diversify affinity‐matured bnAbs, rather than germline precursors.

Overall, despite methodological differences, all three studies converged on the same biological design philosophies. They present compelling data that successful germline‐targeting can go beyond simply recruiting multiple bnAb precursors and can also mature these lineages through repeated rounds of germinal centre selection while progressively altering antigenic selection pressures to favor acquisition of affinity towards a broad panel of native Envs. This represents a major conceptual advance. Previous studies established that precursor recruitment was possible, but whether these cells could survive competition, accumulate sufficient SHM, and mature into authentic bnAbs remained unconfirmed. These studies now show that affinity maturation itself can be deliberately guided. Steichen, Guenaga and Marchitto all demonstrated persistent clonal lineages with extensive intraclonal diversification and heavy‐chain SHM up to or exceeding 10%, culminating in antibodies structurally resembling previously described bnAbs from the V3‐glycan or V2‐apex classes.

The studies also provide several broader insights into bnAb biology. Both Marchitto and Steichen identified multiple bnAb developmental pathways converging on similar structural solutions, suggesting that broadly neutralizing responses may arise from a wider precursor repertoire than originally appreciated. Steichen proposed expanded definitions to identify BG18‐type lineages, and with these recognized that > 75% of animals showed V3‐glycan bnAb expansion. Marchitto's findings suggested that > 25% of V2‐apex binding bnAbs may not utilize the canonical IGHD3‐15*01 RHA1‐class previously assumed to define V2‐apex precursors. Importantly, Marchitto and Steichen also demonstrated that rare bnAb clones induced from the priming immunization can be successively maintained over prolonged periods and even re‐engaged and improved through subsequent vaccinations. These findings are particularly pertinent given they revolve around the exploration of B cells with long HCDR3s, a feature often associated with autoreactivity, and reduced half‐life. However, whether the induced antibodies persist beyond the conclusion of the vaccine regimens remains unknown.

Nevertheless, important challenges remain (Figure 1C). Although these studies represent a significant advancement in the capacity to induce neutralizing bnAb responses against the V2‐apex and V3‐glycan sites, they were not yet able to induce consistent broadly neutralizing serum titres across all macaques. Guenaga and Marchitto were both able to induce some level of autologous neutralization in almost all macaques, but heterologous breadth varied from 20%–70% between animals, with a mean breadth of > 50%. Steichen was able to generate autologous neutralization in 44% of animals, with > 25% of animals showing a breadth of 50%. While the capacity of these studies to induce bnAbs in immunized macaques is a dramatic advancement of the field, it should be noted that the breadth and potency of the most potent bnAbs induced in these studies still fell short of the best naturally occurring human bnAbs [9, 10]. The prolonged immunization schedules, requiring > 5 sequential boosts over extended periods, are unlikely to be practical in the low socioeconomic regions where HIV incidence is highest. However, Marchitto induced bnAbs in 36 weeks, substantially shorter than the 2‐year timeframes used by Guenaga and Steichen, suggesting that further optimisation may further reduce the duration of these regimens. The detailed characterization of the immune response following various immunization strategies presented in all three studies already presents tantalizing implications for how these approaches can be further refined, with certain timepoints, combinations and antigen display strategies yielding more meaningful advances, while other immunization timepoints induced minimal changes to bnAb maturation or breadth. Although the numbers of animals between groups prevented meaningful cross‐study comparisons in efficacy between approaches, the success of these studies strongly implies that this may yield future directions for the field. Despite the considerable advancement provided by these three studies, we cannot yet assume these results will translate to humans. Differences in immunoglobulin gene repertoires and precursor frequency between humans and macaques, particularly for V2‐apex responses, where there is no direct human homologue of IGHD3‐15*01, together with immune tolerance mechanisms acting on long HCDR3‐containing antibodies [2], may substantially influence vaccine performance. Human trials are already underway for the priming immunogens utilized by Marchitto and Steichen, highlighting that further clarity to resolve these concerns may soon be available.

Collectively, these three studies represent a turning point in HIV vaccinology. They provide convergent evidence that germline‐targeting immunization strategies can reproducibly expand rare endogenous bnAb lineages and intentionally guide antibody evolution to generate heterologous neutralizing serum responses in outbred non‐human primates. Although substantial translational hurdles remain, these studies demonstrate the field has progressed beyond simply engaging germline precursors towards the challenges of optimizing the evolutionary pathways that follow. More broadly, these studies establish a conceptual framework that could extend to other HIV epitopes. These studies complement existing evidence that germline‐targeting shows promise for other bnAb classes, including the VRC01‐class CD4 binding site precursor B cells in humans [3]. An important next step may be to determine whether multiple germline‐targeting immunogens can be combined into multivalent vaccination strategies capable of simultaneously initiating several bnAb lineages. Such an approach may ultimately provide the breadth required to overcome HIV's extraordinary antigenic diversity. Together, these studies highlight potential avenues that could be expanded beyond just HIV to develop germline‐targeting vaccine approaches for other rapidly evolving RNA viruses, such as influenza and hepatitis C.

## Author Contributions

**Rowena A. Bull:** conceptualization, writing – original draft, writing – review and editing. **Deborah L. Burnett:** conceptualization, writing – original draft, writing – review and editing.

## Funding

The authors of this work have received funding from UNSW Faculty of Medicine and a UNSW Triple I Seed Grant, support from the Hillcrest Foundation and Perpetual Impact, and the Australian National Health and Medical Research Council (NHMRC) Grants ID 2033069.

## Conflicts of Interest

The authors declare no conflicts of interest.

## Data Availability

Data sharing not applicable to this article as no datasets were generated or analysed during the current study.
